# Texture congruence modulates perceptual bias but not sensitivity to visuotactile stimulation during the rubber hand illusion

**DOI:** 10.3758/s13415-024-01155-2

**Published:** 2024-01-23

**Authors:** Renzo C. Lanfranco, Marie Chancel, H. Henrik Ehrsson

**Affiliations:** 1 Department of Neuroscience, Karolinska Institutet, Solnavägen 9, 171 65 Stockholm, Sweden; 2 Univ. Grenoble Alpes, Univ. Savoie Mont Blanc, CNRS, LPNC, 38000 Grenoble, France

**Keywords:** Body ownership, Rubber hand illusion, Signal detection theory, Psychophysics, Multisensory integration

## Abstract

The sense of body ownership is the feeling that one’s body belongs to oneself. To study body ownership, researchers use bodily illusions, such as the rubber hand illusion (RHI), which involves experiencing a visible rubber hand as part of one’s body when the rubber hand is stroked simultaneously with the hidden real hand. The RHI is based on a combination of vision, touch, and proprioceptive information following the principles of multisensory integration. It has been posited that texture incongruence between rubber hand and real hand weakens the RHI, but the underlying mechanisms remain poorly understood. To investigate this, we recently developed a novel psychophysical RHI paradigm. Based on fitting psychometric functions, we discovered the RHI resulted in shifts in the point of subjective equality when the rubber hand and the real hand were stroked with matching materials. We analysed these datasets further by using signal detection theory analysis, which distinguishes between the participants’ sensitivity to visuotactile stimulation and the associated perceptual bias. We found that texture incongruence influences the RHI’s perceptual bias but not its sensitivity to visuotactile stimulation. We observed that the texture congruence bias effect was the strongest in shorter visuotactile asynchronies (50–100 ms) and weaker in longer asynchronies (200 ms). These results suggest texture-related perceptual bias is most prominent when the illusion's sensitivity is at its lowest. Our findings shed light on the intricate interactions between top-down and bottom-up processes in body ownership, the links between body ownership and multisensory integration, and the impact of texture congruence on the RHI.

**Supplementary Information:**

The online version contains supplementary material available at 10.3758/s13415-024-01155-2.

## Introduction

Body ownership is the subjective experience that one's body is part of oneself. It allows individuals to differentiate their body from other objects in the world (Ehrsson et al., 2004; Tsakiris et al., 2007). This sense of ownership emerges from the integration of visual, proprioceptive, tactile, and other sensory signals alongside previous knowledge about the body and the continuous updating of central body representation (Ehrsson et al., 2004; Ehrsson, 2012; Kilteni et al., 2015; Tsakiris, 2010). Researchers have studied body ownership among healthy participants through bodily illusions, which facilitate manipulation of this experience in ways that would not otherwise be possible. The rubber hand illusion (RHI; Botvinick & Cohen, 1998) is the most widely used bodily illusion (Ehrsson, 2023). It involves synchronously stroking a participant's hidden hand alongside a visible rubber hand, which results in the illusion of owning the rubber hand and experiencing touch on it. The RHI relies on multisensory processes that combine visual and somatosensory information and resolve multisensory conflicts by attributing these signals to a common source (Chancel et al., 2022; Chancel & Ehrsson, 2023; Fang et al., 2019; Samad et al., 2015). The rules governing the RHI reflect the constraints and principles of multisensory perception (Blanke et al., 2015; Ehrsson, 2020). The position, orientation, and synchronous stroking of the rubber hand and real hand are critical to inducing the RHI (Ehrsson et al., 2004; Fuchs et al., 2016; Haans et al., 2008; Kalckert et al., 2019; Kalckert & Ehrsson, 2014; Romano et al., 2015). Moreover, high-level cognitive factors can also influence participants' subjective reports of the RHI at the level of individual differences (Lush et al., 2020; Marotta et al., 2016), although this contribution is overshadowed by the larger effect of multisensory processing and multisensory correlations (Ehrsson et al., 2022; Slater & Ehrsson, 2022). However, knowledge regarding the relative contributions of top-down and bottom-up processes in the sense of body ownership and how the processing of multisensory signals and perceptual biases contribute to this bodily experience remains limited.

Recently, our team developed a new method to objectively and independently measure sensitivity to signal patterns that convey information about body ownership (“body ownership sensitivity”) and perceptual bias in body ownership judgements in the RHI (Lanfranco, Chancel, et al., 2023). This novel approach utilises a signal detection theoretic (SDT) framework (Macmillan & Creelman, 2004) and involves inducing the RHI by using two rubber hands simultaneously, one of which is tapped with varying levels of visuotactile asynchrony with regard to the participant's hand. Participants engage in a two-alternative forced-choice (2AFC) task, as part of which they must indicate which of the two rubber hands feels most like their own (Chancel & Ehrsson, 2020). The 2AFC procedure enables the quantification of difference thresholds for discriminating between two sources of information with regard to body ownership, i.e., the minimal degree of visuotactile asynchrony during the RHI that results in a noticeable perceptual change in illusory hand ownership. This is assessed using the sensitivity (d') metric, which measures the extent to which the feeling of body ownership detects differences between two distinct sources of visuotactile stimulation. Additionally, the 2AFC procedure enables the quantification of the preference (bias) in favour of feeling body ownership with one rubber hand over the other. Because the SDT response bias metric (C) in perceptual illusion paradigms primarily captures perceptual changes (Morgan et al., 1990; Witt et al., 2015, 2016) and 2AFC tasks are naturally robust to response biases (Macmillan & Creelman, 1990, 2004; Peters et al., 2016; Stanislaw & Todorov, 1999), the C metric can be used to quantify illusion-induced perceptual biases (Lanfranco, Chancel et al., 2023).

The results of an SDT analysis that we recently conducted demonstrate that the temporal and spatial correspondence between the visual and tactile stimuli as well as the locations of the participant’s hand and the rubber hands significantly modulate the sensitivity of body ownership to visuotactile stimulation and its bias towards the rubber hand that is associated with the greatest spatial congruence (Lanfranco, Chancel et al., 2023). Importantly, this novel approach allows us to investigate the RHI by using the established framework of SDT, which has been highly successful in many other areas of perception science.

Most previous RHI studies have focused on the spatial and temporal multisensory congruence rules that determine body ownership, that is, whether visual and tactile stimuli occur at the same time and in the same place; however, much less is known about the importance of congruent versus incongruent *contents* of visual and somatosensory information. One form of such multisensory congruence, of which we still know little, is the texture of the materials used to touch the participant’s hand and the rubber hand. The first studies to explore this issue yielded conflicting results. For example, Schütz-Bosbach et al. (2009) found no evidence to indicate that texture congruence affected the RHI even when using materials, such as soft cotton and rough sponge. In contrast, Ward et al. (2015) found that texture incongruence weakened the RHI but only when the difference in texture was significant (e.g., when a pencil vs. a paintbrush was used to stroke the participant's hand and the rubber hand). Similarly, Filippetti et al. (2019) found that texture congruence enhances the RHI irrespective of the pleasantness or unpleasantness associated with the textures used. However, these studies relied on methods, such as questionnaires and proprioceptive drift measurements, which may not always be sufficiently sensitive to detect subtle changes in body ownership. More recently, Chancel and Ehrsson (2020), using the novel 2AFC paradigm described above, found that texture congruence (using plastic and foam materials) influenced body ownership reports. Specifically, these authors found shifts in the point of subjective equality (either rightward or leftward) that indicated a bias in body ownership in favour of the rubber hand touched with the same material as the participant’s real hand. However, the degree to which texture congruence influenced the illusion’s perceptual bias and its sensitivity to visuotactile stimulation was not examined directly, nor were the potential interactions between sensitivity and bias explored.

To address this question, we conducted an SDT analysis using the datasets collected for Chancel and Ehrsson’s (2020) Experiment 2. Our goal was to determine whether the congruence of textures between the materials in contact with the participant’s hand and those in contact with the rubber hands influences body ownership in the RHI by modulating two key aspects: body ownership sensitivity to visuotactile asynchrony (referred to as body ownership sensitivity) and the RHI’s preference for a rubber hand stimulated by an object with matching tactile material properties (referred to as perceptual bias). If texture congruence promotes the bottom-up processing of the temporal properties of visual and tactile signals, we would expect increased sensitivity to visuotactile asynchrony to be observed in trials in which the visual and tactile materials are congruent. In this context, sensitivity (d') quantifies the ability of body ownership to distinguish between signal (i.e., the rubber hand is stroked synchronously with the real hand) and noise trials (i.e., the rubber hand is stroked asynchronously). Conversely, if texture congruence exerts its influence through top-down processes or contextual cues extracted from the sensory environment that facilitate the integration of texture-matching visual and somatosensory information, possibly based on previous multisensory experiences of visual and tactile cues pertaining to texture, we would expect to observe a stronger sense of ownership over the rubber hand when it is touched with a texture that matches the texture touching the participant's real hand. In this case, this feeling can be quantified as a bias towards experiencing a more robust illusion with either the left or right rubber hand that receives such congruent texture stimulation.

Finally, because previous studies have indicated that the influence of perceptual biases is most pronounced under conditions in which sensory signals are weak or ambiguous (Lanfranco, Canales-Johnson, et al., 2022; Lanfranco, Chancel et al., 2023; Lanfranco, Rabagliati et al., 2023; Lanfranco, Stein et al., 2022), we also explored the possible effects of interactions between the degree of visuotactile asynchrony and texture congruence on perceptual bias.

## Method

### Participants

Chancel and Ehrsson (2020) recruited 30 naïve participants but included data only from those who experienced a vivid RHI, because it is necessary to experience the illusion to perform the task, and previous studies have reported that ~20–25% of such participants fail to affirm the illusion (Kalckert & Ehrsson, 2014). In this inclusion test, each participant sat in front of a table with their right hand resting on a support. Fifteen centimeters above their hand, there was a small table with a life-sized prosthetic right hand (model 30916-R, Fillauer®, filled with plaster) placed in the same orientation as the hidden real hand. Participants kept their eyes fixated on the rubber hand while the experimenter stroked the rubber hand and the real hand for 12 s, synchronising the timing of the stroking as closely as possible. Each stroke lasted for 1 s. The strokes were applied to different locations on the index fingers, at a frequency of 0.5 Hz. Next, participants were asked to complete the same RHI questionnaire used by Botvinick and Cohen (1998). The inclusion criteria consisted of: (a) mean score on the ownership statements (Q1–3) greater than 1, and (b) the difference between this mean score and the mean score of the control items (Q4–9) greater than one.

We employed the same selection criteria: eight participants did not meet the minimum threshold for a strong RHI, and one dataset was excluded due to missing data, resulting in a final sample of 21 participants (12 females; M_age_ = 24.2, SD_age_ = 3.76). Because the original study was the first of its kind and effect sizes were unknown, Chancel and Ehrsson (2020) did not perform a power analysis; instead, they chose a sample size that matched typical sample sizes from previous RHI studies (Brozzoli et al., 2012; Guterstam et al., 2011; Preston, 2013; Rohde et al., 2011).

The study was approved by the Ethical Review Authority, and all participants provided informed consent in accordance with the Declaration of Helsinki. Participants were paid 300 SEK for their participation.

### Stimuli and apparatus

**Fig. 1:**
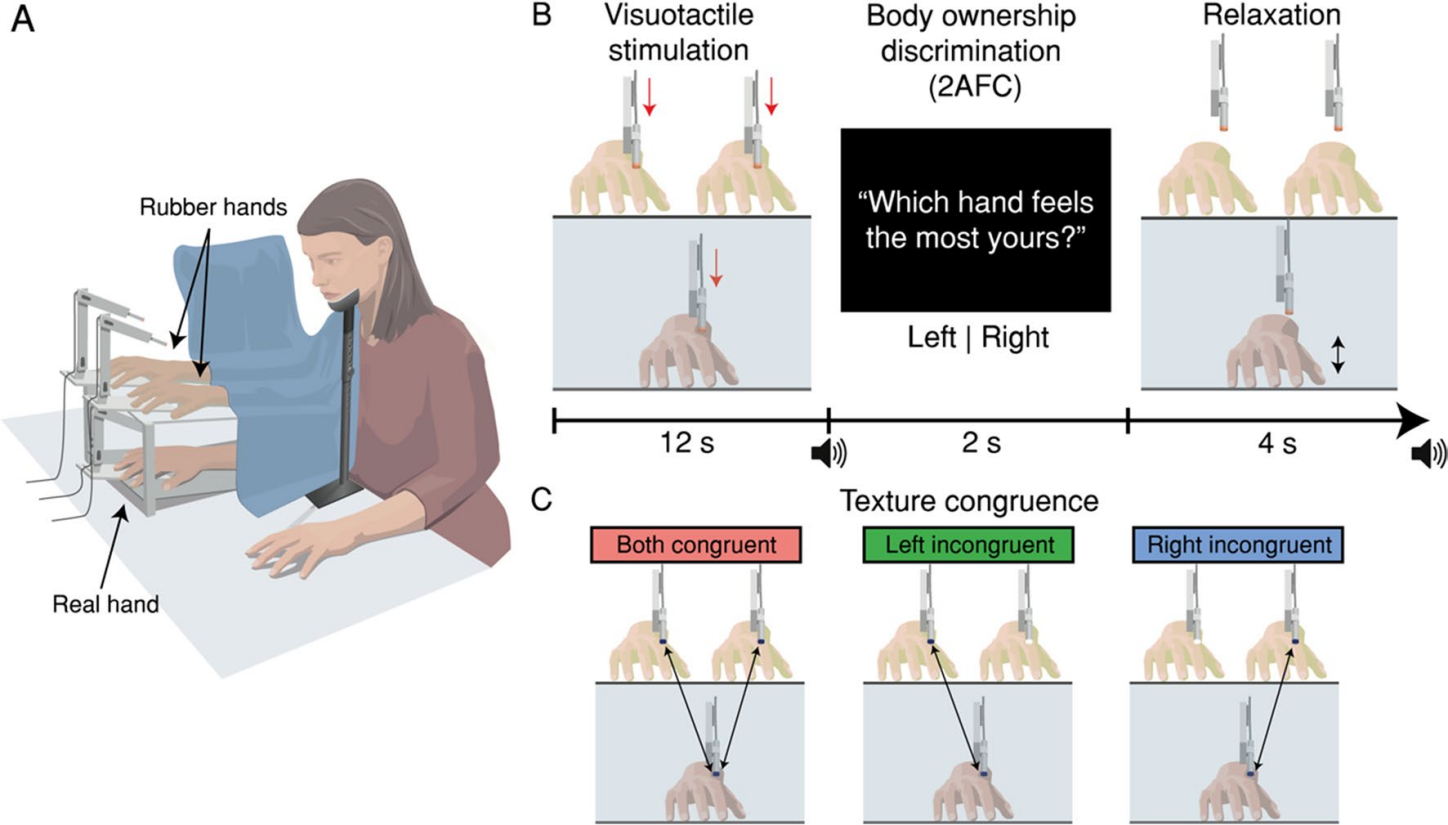
**(A) Experimental setup.** Two robot arms apply touches to both rubber hands placed on top of the table, and one robot arm applies touches to the participant’s real hand, which is hidden below the platform. A white fixation dot is located halfway between the two rubber hands. (**B**) Trial schematics. The robot arms tap the rubber hands and the real hand with different degrees of asynchrony between the rubber hands; crucially, one rubber hand is always synchronously tapped with the real hand, which is the condition that we know produces the strongest RHI. Next, an auditory cue informs participants that they must verbally indicate which hand felt most like their own (left or right). An auditory cue informs them when the next trial is about to begin. (**C**) Texture congruency conditions. The materials touching the rubber hands and the real hand are manipulated such that the material touching the rubber hands and the real hands are the same (both textures congruent), such that only the material touching the right rubber hand and the real hand are the same (left texture incongruent), or such that only the material touching the left rubber hand and the real hand are the same (right texture incongruent). The arrows and colour of the robot arms’ tips represent the texture congruence conditions. The robot arms’ tips are represented in different colours for didactic purposes, but they did not differ visually in the setup. Illustration by Mattias Karlén.

Participants placed their right hands, palm down, on a flat surface beneath a table, 30 cm from the body midline. Two identical rubber hands were positioned on the table and tilted 30° upwards at the front; these rubber hands were placed next to each other at the same distance from the real hand (Fig. 1A). This setup enabled the RHI to be induced on both rubber hands simultaneously (Ehrsson, 2009; Fan et al., 2021; Lanfranco, Chancel et al., 2023), and a white circular fixation mark was placed between the two rubber hands. Participants’ heads were kept steady using a chin rest, and their right arm was supported with an elbow rest (Ergorest Oy®, Finland) to ensure relaxation.

Tactile stimuli were delivered to the rubber and real hands by three robot arms, each of which consisted of two 17-cm-long and 3-cm-wide metal pieces and a metal slab (10 × 20 cm). Two HS-7950TH UltraTorque servos powered the joint that connected the metal pieces, while the proximal and support parts were powered by another servo. A touch probe (7-mm diameter) attached to the end of the distal metal piece touched the hands during the stimulation procedure, and E3X-HD41 fibre sensors (OMRON®, Netherlands) registered the exact timing of the taps by measuring the time that the red laser light took to bounce back when the touch probes made contact with the hands. Two different materials were utilised as touch probes on the robot arms: firm plastic and polyethylene foam. Both types of probes shared identical dimensions and shapes, specifically a cylindrical form with a 7-mm diameter. Additionally, the probes were designed to exhibit a degree of flexibility, allowing for slight bending upon contact with either the rubber hands or the real hand. Therefore, the distinction between these stimuli pertained to their respective material textures. The applied and theoretical degrees of asynchrony were similar, as confirmed by the lasers. To minimise distractions, participants wore earphones and listened to individually adjusted white noise levels.

### Procedure

Participants were instructed to focus on the fixation mark while the robot arms repeatedly tapped their index fingers six times over a period of 12 s. Five different locations were tapped in a randomised order that was congruent across hands to avoid skin irritation (proximal to the nail on the distal phalanx, on the distal interphalangeal joint, on the middle phalanx, on the proximal interphalangeal joint, or on the proximal phalanx). After an auditory cue, participants indicated which rubber hand felt most like their own as part of a 2AFC task. They were then asked to relax and wiggle their fingers to break the illusion and prevent muscle numbness before the next trial began; another auditory cue indicated the start of the next trial (Fig. 1B). Seven degrees of asynchrony were applied, with either the left or right hand touched after a delay (0, 50, 100, or 200 ms; in the 0-ms condition, the three hands were tapped synchronously). The congruence between the materials touching the rubber hands and the real hand (texture congruence) was manipulated by attaching either firm plastic or polyethylene foam to the robot hands’ tips, which entailed differences in tactile roughness and hardness (i.e., smooth vs. greater surface friction and soft vs. hard material; Fig. 1C). A total of 504 semirandomly ordered trials were conducted, which were evenly distributed over three experimental factors: degree of asynchrony between taps, type of material touching the real hand, and congruence between the material held by the robots tapping the real hand and the rubber hands (both textures congruent, left texture incongruent, right texture incongruent). The six different conditions were tested in separate blocks, which were presented in a randomised order across participants. Prior studies have shown that RHI can be reliably induced in 10 s (Chancel & Ehrsson, 2020; Ehrsson et al., 2004; Lloyd, 2007), which was confirmed in pilot sessions. The use of earphones to provide white noise helped to prevent distraction from the noise produced by the robot arms. For a more detailed description of the procedures, see Chancel and Ehrsson (2020).

## Transparency and openness

We report how our sample size was determined, all data exclusions, all manipulations, and all measures in the study, and we follow Journal Article Reporting Standards (JARS; Kazak, 2018). Data and analyses are publicly available on the Open Science Framework: https://osf.io/spkvu/. Data were analysed by using MATLAB 2022b (Mathworks, Inc), JASP (JASP Team, 2023), and R (version 4.1.0) software. This study design and the corresponding analysis were not preregistered.

### Analysis

To evaluate changes in body ownership sensitivity to visuotactile signals and perceptual bias across different conditions of stimulation asynchrony and texture congruence, we employed SDT analysis. For each participant, we calculated all measures for each combination of factors. To determine body ownership sensitivity or d', we used the 2AFC formula, ${d}^{\prime } ownership=\left(\frac{1}{\sqrt{2}}\right)\left(Z(Hit)-Z(FA)\right)$, where hits were defined as trials in which the participant reported that the right rubber hand felt most like their own when the right rubber hand was synchronously tapped with the real hand; similarly, false alarms (FAs) were defined as trials in which the participant reported that the right rubber hand felt most like their own when the left rubber hand was synchronously tapped with the real hand (Macmillan & Creelman, 2004). Thus, d’ quantifies the extent to which participants’ feelings of body ownership detect differences in visuotactile stimulation across different degrees of visuotactile asynchrony while controlling for biases. Perceptual bias towards the left or right rubber hand was determined by using the 2AFC formula for decision criterion, $Crubber\ hand=-\left(\frac{1}{2}\right)\left(Z(Hit)+Z(FA)\right)$. In this context, positive and negative values indicate a bias towards claiming illusory ownership over the left and right rubber hands, respectively. Thus, C quantifies the direction and magnitude of the subjective feeling of ownership with regard to the left or right rubber hand across conditions. We used repeated-measures analysis of variance (ANOVA) to analyse body ownership sensitivity and perceptual bias and applied Greenhouse–Geisser correction whenever Mauchly's test indicated violation of the sphericity assumption. For significant interactions (*p* < .05), we conducted post hoc pairwise comparisons, Holm–Bonferroni-corrected, based on the pooled variance in the ANOVA model using estimated marginal means. Because null results do not necessarily indicate the absence of an effect, we also assessed the data by using a Bayesian repeated-measures ANOVA (Rouder et al., 2012; van den Bergh et al., 2023) to determine the extent to which the data supported each alternative hypothesis model of interest. We calculated Bayes factors to assess the strength of evidence for the null and alternative hypotheses. To accomplish this, we employed a uniform prior distribution with r-scale coefficients of 0.5 width for fixed effects, 1 for random effects, and 0.354 for covariates in Bayesian repeated-measures ANOVA models, and a Cauchy prior distribution centred around zero with a width parameter of 0.707 for Bayesian *t*-tests; these parameters are widely accepted as default parameters in the absence of prior real data. Sensitivity analyses of priors were run to assess the robustness of the Bayesian repeated-measures ANOVA’s default prior distribution, where wider (r-scale prior width of 0.8) and narrower (r-scale prior width of 0.2) prior distributions were employed (Depaoli & van de Schoot, 2017; van den Bergh et al., 2023). We excluded trials from the zero-asynchrony condition from the analysis since they could not be classified as hits or FAs.

## Results

### Body ownership sensitivity

**Fig. 2:**
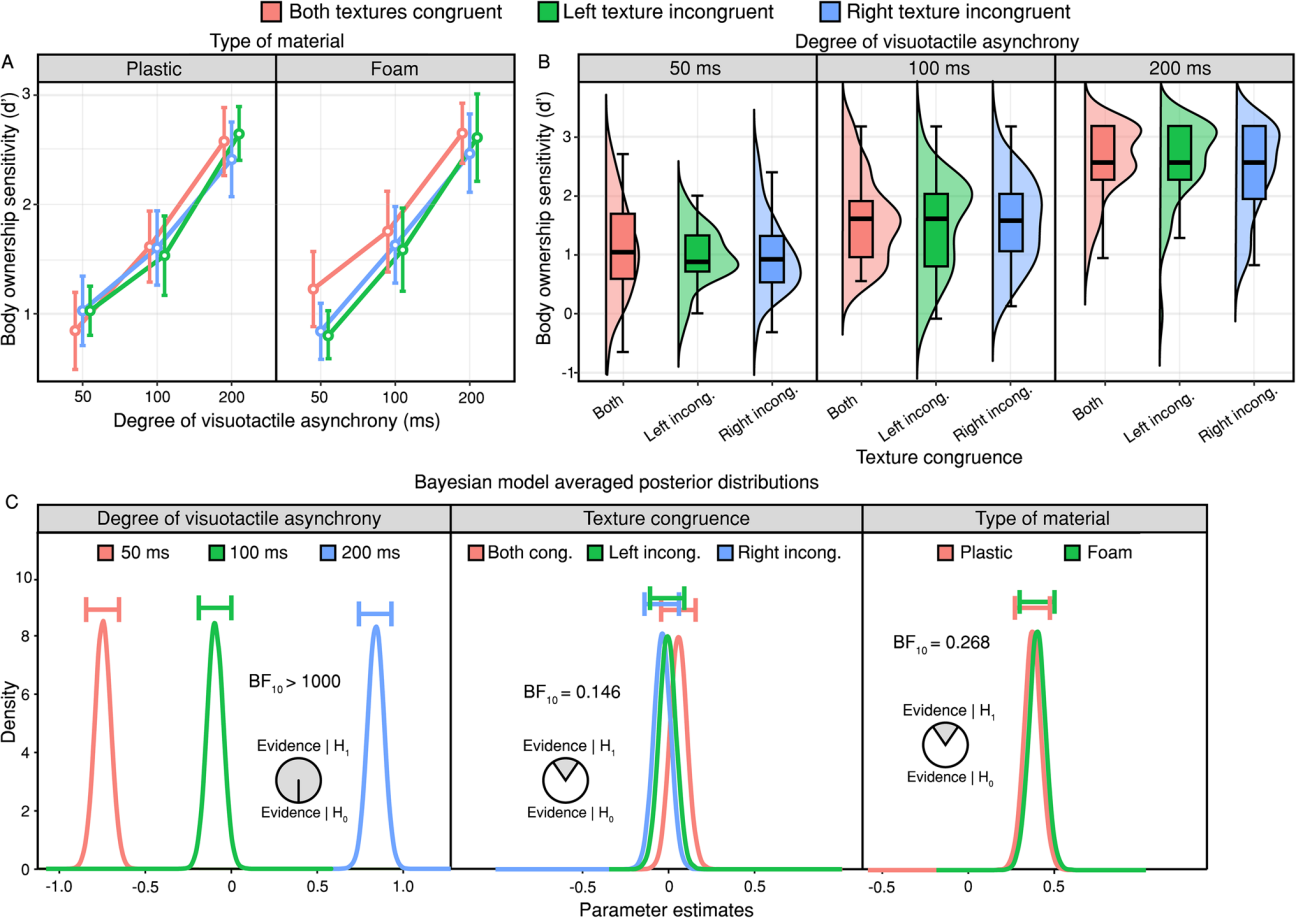
**Body ownership sensitivity results.** (**A–B**) Body ownership d’ increased with increasing asynchrony, but it was unaffected by incongruencies between the materials tapping the rubber hands and the material tapping the real hand (texture incongruence). Error bars represent 95% confidence intervals. (**C**) Posterior distributions of the effects of degree of asynchrony (left), texture congruence (centre), and type of material (right), excluding higher-order interactions. The evidence provides extreme support for the alternative hypothesis model of degree of asynchrony and moderate support for both the null hypothesis model of texture congruence and the null hypothesis model of type of material. The horizontal bars above each density represent 95% credible intervals

By-condition body ownership d’ scores were entered into a 3 (degree of asynchrony: 50, 100, 200 ms) × 3 (texture congruence: both textures congruent, left texture incongruent, right texture incongruent) × 2 (type of material: plastic, foam) repeated-measures ANOVA. We only found a significant main effect of the degree of asynchrony (*F*_(2, 40)_ = 240.15, *p* < .001, ηp^2^ = .923), which replicated the main finding of Lanfranco, Chancel et al. (2023). However, no other effects (*all p* > .422) or interactions (*all p* > .144) reached significance (Figs. 2A–B). Bayes factor analysis provided extreme support for the alternative hypothesis model of degree of asynchrony (*BF*_10_ > 100) and moderate support for both the null hypothesis models of texture congruence (*BF*_10_ = 0.142) and type of material (*BF*_10_ = 0.244). Model*-*averaged posterior distributions of specific effects are shown (Fig. 2C). See Table 1 for the descriptive statistics.

**Table 1:** Descriptive statistics

Material	Texture congruence	Degree of asynchrony (ms)	Body ownership sensitivity		Perceptual bias	
			M	SD	M	SD
Plastic	Both congruent	50	0.847	0.747	0.075	0.608
		100	1.614	0.682	0.088	0.479
		200	2.578	0.646	0.012	0.378
	Left incongruent	50	1.029	0.665	0.516	0.683
		100	1.601	0.725	0.456	0.531
		200	2.412	0.731	0.286	0.323
	Right incongruent	50	1.029	0.472	-0.436	0.630
		100	1.533	0.772	-0.429	0.516
		200	2.645	0.531	-0.264	0.397
Foam	Both congruent	50	1.227	0.722	0.039	0.800
		100	1.755	0.781	0.053	0.649
		200	2.652	0.581	-0.142	0.373
	Left incongruent	50	0.840	0.540	0.207	0.703
		100	1.629	0.743	0.428	0.653
		200	2.465	0.776	0.143	0.366
	Right incongruent	50	0.801	0.464	-0.374	0.820
		100	1.585	0.807	-0.370	0.725
		200	2.612	0.841	-0.268	0.623

Means and standard deviation scores for body ownership sensitivity (d’) and perceptual bias across each repeated-measures factor, namely type of material, texture congruence, and degree of visuotactile asynchrony. Individual scores for each factor can be accessed in the Open Science Framework repository: https://osf.io/spkvu/.

To assess the robustness of our Bayes factor results, we conducted a sensitivity analysis of priors, manipulating the r-scale coefficient of the prior distribution for fixed effects. This exploration was designed to determine if such manipulations lead to meaningful changes in Bayes factors. Our results indicate that both wider prior distributions (r-scale prior width of 0.8) and narrower ones (r-scale prior width of 0.2) compared with our default prior (r-scale prior width of 0.5) produce similar results. When employing a wider prior distribution, we observed extreme support for the alternative hypothesis model of the degree of asynchrony (*BF*_10_ > 100), whereas we found strong and moderate support for the null hypothesis models of texture congruence (*BF*_10_ = 0.068) and type of material (*BF*_10_ = 0.174), respectively. Similarly, utilising a narrower prior distribution resulted in extreme support for the alternative hypothesis model of the degree of asynchrony (*BF*_10_ > 100), along with anecdotal and strong support for the null hypothesis models of texture congruence (*BF*_10_ = 0.413) and type of material (*BF*_10_ = 0.012), respectively. These results support the robustness of our default prior parameters, indicating that variations in the r-scale coefficient do not meaningfully affect Bayes factors.

These results indicate that sensitivity to body ownership signals increases with increasing visuotactile asynchrony and that this process is unaffected by texture incongruence. With regard to the normality and nonparametric tests, see the Supplementary Material.

### Perceptual bias

**Fig. 3:**
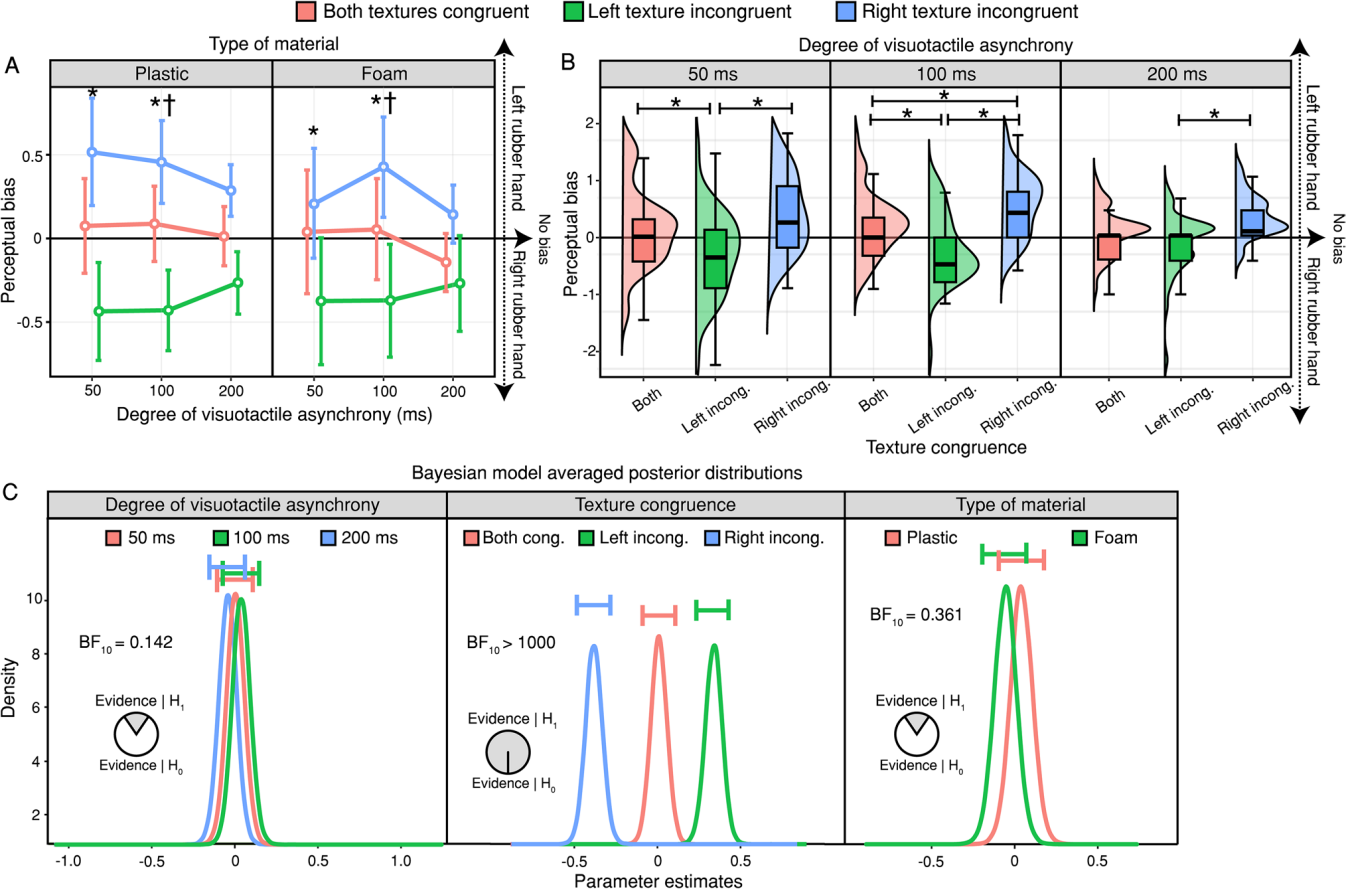
**Perceptual bias results.** (**A–B**) Participants exhibited a bias in favour of the rubber hand that was tapped with the same material used on the real hand, but only when the other rubber hand was tapped with a different material. However, when the degree of asynchrony was the highest (200 ms), neither the comparison between the “Both textures congruent” and “Left texture incongruent” conditions nor the comparison between the “Both textures congruent” and “Right texture incongruent” conditions indicated significant differences. Asterisks denote a significant difference between the “Both textures congruent” and “Left texture incongruent” conditions. Daggers denote significant differences between the “Both textures congruent” and “Right texture incongruent” conditions. Error bars represent 95% confidence intervals. (**C**) Posterior distributions of the effects of (left) degree of asynchrony, (centre) texture congruence, and (right) type of material, excluding higher-order interactions. The evidence provides substantial support for the null hypothesis model of the degree of asynchrony, extreme support for the alternative hypothesis model of texture congruence, and anecdotal support for the null hypothesis model of type of material. The horizontal bars above each density represent 95% credible intervals

By-condition perceptual bias scores were entered into a 3 (degree of asynchrony: 50, 100, 200 ms) × 3 (texture congruence: both textures congruent, left texture incongruent, right texture incongruent) × 2 (type of material: plastic, foam) repeated-measures ANOVA. We found a significant main effect of texture congruence (*F*_(1.33, 26.65)_ = 39.715, *p* < .001, ηp^2^ = .665)*,* thus indicating that texture incongruence modulated perceptual bias (Figs. 3A–B); the main effects of asynchrony and material were not significant; in all cases, *p* > .419). Post hoc comparisons revealed significant differences between the “Both textures congruent” and “Left texture incongruent” conditions (*t*_(20)_ =  − 4.829, *p* < .001, *d* =  − 1.054), between the “Both textures congruent” and “Right texture incongruent” conditions (*t*_(20)_ = 4.072, *p* < .001, *d* = 0.889), and between the “Left texture incongruent” and “Right texture incongruent” conditions (*t*_(20)_ = 8.902, *p* < .001, *d* = 1.942).

Only the interaction between the degree of asynchrony and texture congruence reached significance (*F*_(4, 80)_ = 2.649, *p* = .039, ηp^2^ = .117). Post hoc comparisons revealed significant differences between the “Both textures congruent” and “Right texture incongruent” conditions at 100 ms (*t*_(20)_ = 3.494, *p* < .015, *d* = 1.009) but not at 50 ms (*t*_(20)_ = 2.861, *p* = .098, *d* = 0.499) or 200 ms of asynchrony (*t*_(20)_ = 2.629, *p* = .139, *d* = 0.768). They also revealed differences between the “Both textures congruent” and “Left texture incongruent” conditions at 50 ms (*t*_(20)_ =  − 4.343, *p* < .001, *d* =  − 1.151) and 100 ms (*t*_(20)_ =  − 4.420, *p* < .001, *d* =  − 1.370) but not at 200 ms of asynchrony (*t*_(20)_ =  − 1.890, *p* = .678, *d* =  − 0.520). As Fig. 3B shows, all comparisons between the “Left texture incongruent” and “Right texture incongruent” conditions were significant: 50 ms (*t*_(20)_ = 7.203, *p* < .001, *d* = 0.942), 100 ms (*t*_(20)_ = 7.914, *p* < .001, *d* = 1.762), and 200 ms (*t*_(20)_ = 4.519, *p* < .001, *d* = 1.128).

To determine whether participants exhibited a bias in the “Both textures congruent” condition, we performed a series of one-sample *t* tests against zero on the perceptual bias scores obtained for that condition, which did not differ significantly from zero (*all p* > .095)*.* Bayes factor analysis provided moderate support for the null hypothesis model (3.211 ≤ *BF*_0 +_  ≤ 4.352), except when the material used was foam and the degree of asynchrony with which the rubber hands were tapped was 200 ms, in which case it provided only anecdotal support for the null hypothesis (*BF*_0 +_  = 1.203).

Bayes factor analysis provided extreme support for the alternative hypothesis model of texture congruence (*BF*_10_ > 100) and strong and anecdotal support for the null hypothesis models of degree of asynchrony (*BF*_10_ = 0.136) and type of material (*BF*_10_ = 0.335), respectively. Model*-*averaged posterior distributions of specific effects are shown (Fig. 3C).

To assess the robustness of our Bayes factor results, we conducted another sensitivity analysis of priors. Both wider prior distributions (r-scale prior width of 0.8) and narrower ones (r-scale prior width of 0.2) yielded Bayes factors similar to those obtained with our default prior (r-scale prior width of 0.5). Using a wider prior distribution, we observed strong and moderate support for the null hypothesis models of the degree of asynchrony (*BF*_10_ = 0.065) and type of material (*BF*_10_ = 0.259), respectively, along with extreme support for the alternative hypothesis model of texture congruence (*BF*_10_ > 100). Similarly, with a narrower prior distribution, we found strong and anecdotal support for the null hypothesis models of the degree of asynchrony (*BF*_10_ = 0.023) and type of material (*BF*_10_ = 0.623), respectively, and extreme support for the alternative hypothesis model of texture congruence (*BF*_10_ > 100). These results support the robustness of our default prior parameters.

These results indicate that the differences observed in perceptual bias between texture congruence conditions decreased as the degree of visuotactile asynchrony increased. Importantly, at 200 ms of asynchrony, neither texture-incongruent condition differed significantly from the “Both textures congruent” condition, which may suggest that the effect of texture congruence on body ownership relies less on top-down factors as sensory evidence increases. In other words, when the degree of asynchrony is 200 ms and body ownership is thus significantly weakened, the perceptual (rubber hand) bias that leads to the perception of visual and somatosensory events involving objects with similar texture properties as a combined experience no longer has a significant impact on the ownership perception of the stimulated rubber hand.

## Discussion

Does texture congruence between the materials touching the (seen but unfelt) rubber hands and the (felt but unseen) real hand modulate body ownership in the RHI? Previous studies based on questionnaires and proprioceptive drift measurements have reported contradictory findings (Schütz-Bosbach et al., 2009; Ward et al., 2015). More recently, Chancel and Ehrsson (2020) used a more sensitive 2AFC discrimination task to reveal that texture congruence enhances body ownership. To investigate whether this effect was driven primarily by an increase in sensitivity to visuotactile signals or by a perceptual bias towards objects with matching material texture properties, we employed SDT analysis. Our results suggest that texture congruence modulates the RHI by inducing a perceptual bias: participants felt ownership more often with the rubber hand that was tapped with the same material as their hidden real hand. Notably, this preference diminishes with increasing visuotactile stimulation asynchronies, suggesting that reliance on the textural congruence bias decreases as multisensory bottom-up evidence against body ownership increases.

Our findings suggest that the effect of a congruent or incongruent texture in the RHI is driven primarily by a perceptual bias toward congruent textures, which is presumably related to top-down processes. Such top-down processes could include prior lifetime experiences of manually interacting with objects composed of different materials; plastic and foam, which were used in the current study, are two such common materials. Top-down processes could also involve tactile texture expectations, which are automatically generated by the visual impressions of seeing the objects moving towards and touching the skin of the rubber hand (Chancel et al., 2021); when these tactile expectations do not match the tactile sensory signals, a bias against integrating visual and somatosensory information emerges, and fewer rubber hand ownership judgements are made. The notion that the texture bias is perceptual in nature and is related to top-down processes in perceptual inference is in line with the finding of an interaction between asynchrony and bias, such that the bias effect is no longer significant when the degree of asynchrony reaches 200 ms. At a degree of asynchrony of 200 ms, the sensory evidence favouring multisensory combination for the synchronously tapped rubber hand is so strong that the bias towards ownership perception for the rubber hand receiving texture-matching visuotactile stimulation no longer makes a significant contribution. We also think that it is very unlikely that the current perceptual bias effect can be explained solely by reference to post-perceptual cognitive factors (e.g., reasoning, expectations), because such high-level effects would presumably influence all levels of visuotactile asynchrony. Moreover, the participants were never informed about the fact that different objects were used, they did not observe the experimenter changing these objects between experimental trial runs, and they never saw which material was touching their real hand; furthermore, postexperimental interviews suggested that the participants did not spontaneously notice or reflect on the probe materials used for the touch by the robots. Thus, our interpretation posits that the texture congruence effect most likely results from perceptual bias related to automatic top-down and/or bottom-up contextual processes.

Our findings also show that body ownership sensitivity is remarkably precise, exhibiting above-chance performance at only 50 ms of stimulation asynchrony; this finding replicates the main finding of a recent publication using our SDT method (Lanfranco, Chancel et al., 2023). Furthermore, body ownership sensitivity was unaffected by object texture information, and the evidence in favour of this finding is very strong in our Bayesian analysis. However, the current data cannot rule out the possibility that using increasing incongruence in terms of objects’ texture, curvature, hardness, and other material properties further might influence the illusion’s sensitivity to visuotactile asynchrony. Another interesting direction for future studies is to directly test the sensitivity of bottom-up processing of texture information by conducting experiments based on a subtle and stepwise experimental manipulation of the degree of texture incongruence (similar to the current stepwise manipulation of visuotactile asynchrony) in fully synchronous conditions and to analyse possible changes in body ownership sensitivity or the lack thereof.

Other congruence effects could be investigated in further detail using the current 2AFC SDT approach. For example, tactile affective congruence (Filippetti et al., 2019) and visuothermal congruence (Crucianelli & Ehrsson, 2022) of the objects touching the rubber hand and the real hand have also been reported to enhance the RHI. Similarly, congruence in the humanoid shape and form of the rubber hand as compared with the participant’s real hand also influences the RHI (Tsakiris et al., 2010). Our method could be used to determine whether these congruence effects are driven primarily by changes in sensitivity to visuotactile and other bottom-up sensory processing or by perceptual biases caused by top-down processes.

A requirement of our paradigm is that participants must experience the RHI; otherwise, psychometric functions cannot be fitted to the data, nor can SDT analyses be conducted. As in the case of many illusions, not everyone is susceptible to the RHI. Current estimates indicate that approximately 60–80% of participants affirm the illusion in the classic synchronous visuotactile stimulation condition according to questionnaire results (Ehrsson, 2023; Kalckert & Ehrsson, 2014; Reader et al., 2021). Consequently, only participants who experienced the RHI with sufficient intensity were eligible to participate in the study from which the current data were generated. As a result, our findings might not be applicable to individuals who are unable to perceive bodily illusions. Future research should investigate whether the results and conclusions about human bodily perception derived from bodily illusion paradigms can be generalised to a wider population.

## Conclusions

Our results contribute to resolving a debate in the field of body ownership research by showing that texture congruence has an effect on the RHI, as highlighted by Chancel and Ehrsson (2020), Filippetti et al. (2019), and Ward et al. (2015), but that it is driven primarily by a perceptual bias related to texture congruence. While texture congruence enhances the RHI, it does not modulate sensitivity to the synchronicity or asynchronicity of the correlated visuotactile signals, thereby suggesting that either larger differences in texture are necessary to achieve this effect or that texture congruence modulates the RHI exclusively through perceptual bias.

### Supplementary information

ESM 1 (PDF 158 kb)
