# Supplementary material for: Texture congruence modulates perceptual bias but not sensitivity to visuotactile stimulation during the rubber hand illusion
Source: Cogn Affect Behav Neurosci. 2024 Jan 23;24(1):100–10. doi: 10.3758/s13415-024-01155-2 (PMC10827897; doi:10.3758/s13415-024-01155-2)
Supplement: Supplementary file 1 — (PDF 158 kb) [file 13415_2024_1155_MOESM1_ESM.docx]

**Supplementary Material**

**Texture congruence modulates perceptual bias but not sensitivity to visuotactile stimulation during the rubber hand illusion**

**Normality tests and non-parametric tests**

We assessed normality by using the Shapiro-Wilk test (recommended for samples of less than 50 individuals; Mishra et al., 2019), and by visually inspecting quantile-quantile (Q-Q) plots for each condition, separately.

For the analysis of body ownership sensitivity, only the 200 ms conditions departed from normality: when the three hands were tapped by plastic $\left( W=0.84, p=.003 \right)$; when only the left hand was tapped by foam $\left( W=0.88, p=.015 \right)$; when only the participant’s hand was tapped by foam $\left( W=0.86, p=.006 \right)$; when all the hands were tapped by foam $\left( W=0.804, p<.001 \right)$; when only the left rubber hand was tapped by plastic $\left( W=0.845, p=.004 \right)$; and when only the participant’s hand was tapped by plastic $\left( W=0.737, p<.001 \right)$. These conclusions were supported by Q-Q plots. Therefore, we ran Friedman tests to assess whether the data of these conditions, which showed equivalent results to those reported by parametric tests. No other condition showed a significant departure from normality.

For the analysis of perceptual bias, only the 100 ms condition when only the participant’s hand was tapped by foam departed from normality $\left( W=0.881, p=.015 \right)$, and the following 200 ms conditions departed from normality: when only the left hand was tapped by foam $\left( W=0.832, p=.002 \right)$; when all the hands were tapped by foam $\left( W=0.883, p=.017 \right)$; and when only the participant’s hand was tapped by plastic $\left( W=0.79, p<.001 \right)$. These conclusions were supported by Q-Q plots. Therefore, we ran Friedman tests to assess whether the data of these conditions, which showed equivalent results to those reported by parametric tests. No other condition showed a significant departure from normality.

**References**

Mishra, P., Pandey, C. M., Singh, U., Gupta, A., Sahu, C., & Keshri, A. (2019). Descriptive statistics and normality tests for statistical data. *Annals of Cardiac Anaesthesia*, *22*(1), 67. https://doi.org/10.4103/aca.ACA_157_18
